#  A comparison of medical education in Germany and the United States: from applying to medical school to the beginnings of residency

**DOI:** 10.3205/000256

**Published:** 2017-09-25

**Authors:** Dmitry Zavlin, Kevin T. Jubbal, Jonas G. Noé, Bernd Gansbacher

**Affiliations:** 1 Institute for Reconstructive Surgery, Houston Methodist Hospital, Weill Cornell Medicine, Houston, TX, USA; 2 Department of Plastic Surgery, Loma Linda University Medical Center, Loma Linda, CA, USA; 3 Department of Medicine, Washington University in St. Louis, St. Louis, MO, USA; 4 Institute of Molecular Immunology & Experimental Oncology, Technical University Munich, Munich, Germany

**Keywords:** medical school, education, physician, Germany, United States

## Abstract

Both Germany and the United States of America have a long tradition of science and medical excellence reaching back as far as the nineteenth century. The same tribute must be paid to the medical educational system in both countries. Despite significant initial similarities and cross-inspiration, the paths from enrolling in a medical university to graduating as a medical doctor in Germany and the US seem to have become much different. To fill a void in literature, the authors’ objective therefore is to delineate both structures of medical education in an up-to-date review and examine their current differences and similarities. Recent medical publications, legal guidelines of governmental or official organizations, articles in media, as well as the authors’ personal experiences are used as sources of this report.

Tuition loans of over $200,000 are not uncommon for students in the US after graduating from medical schools, which are often private institutions. In Germany, however, the vast majority of medical universities are tax-funded and, for this reason, free of tuition. Significant differences and surprisingly multiple similarities exist between these two systems, despite one depending on government and the other on private organizations. Germany currently employs an integrated medical curriculum that typically begins right after high school and consists of a 2-year long pre-clinical segment teaching basic sciences and a 4-year clinical segment leading medical students to the practical aspects of medicine. On the other hand, the US education is a two-stage process. After successful completion of a Bachelor’s degree in college, an American student goes through a 4-year medical program encompassing 2 years of basic science and 2 years of clinical training. In this review, we will address some of these similarities and major differences.

## Zusammenfassung

Deutschland und die Vereinigten Staaten von Amerika haben beide eine lange Tradition der Naturwissenschaft und medizinischen Exzellenz, die bis weit in das neunzehnte Jahrhundert zurückreicht. Den gleichen Tribut muss man den medizinischen Ausbildungssystemen beider Länder zollen. Trotz zu Beginn bedeutsamer Ähnlichkeiten und gewisser Querinspiration scheinen sich die Wege von der Immatrikulation an einer medizinischen Fakultät bis zum Studienabschluss als Arzt in Deutschland und den USA getrennt zu haben. Um eine Lücke in der Fachliteratur zu schließen, ist das Ziel der Autoren, die beiden Strukturen der medizinischen Ausbildung mittels einer aktuellen Übersichtsschrift darzustellen und deren Unterschiede und Gemeinsamkeiten zu untersuchen. Die neusten medizinischen Publikationen, verbindliche Richtlinien von amtlichen oder offiziellen Organisationen, Artikel in der Presse, aber auch die persönlichen Erfahrungen der Autoren dienen als Quellen für diese Arbeit.

Studienkredite von über $200.000 sind nicht selten für Studenten in den USA nach deren Abschluss an einer medizinischen Hochschule, die meist in privatem Eigentum ist. In Deutschland dagegen ist die große Mehrheit der Universitäten mit medizinischen Fakultäten in öffentlicher Hand, aus Steuern finanziert und deshalb frei von Studiengebühren. Signifikante Unterschiede doch auch überraschenderweise eine Reihe von Ähnlichkeiten existieren zwischen den Systemen der zwei Länder, obwohl eines von privaten Einrichtungen und das andere von staatlichen Hochschulen abhängig ist. Deutschland verwendet aktuell ein ganzheitliches medizinisches Curriculum, das klassischerweise direkt nach dem Abitur beginnt und aus zwei Jahren vorklinischer und vier Jahren klinischer Ausbildung besteht, wobei letzteres die Studenten an die praktischen Aspekte der Medizin heranführen soll. Auf der anderen Seite herrscht in den USA ein zweistufiger Ausbildungsprozess. Nach erfolgreichem Erreichen eines Bachelorgrads im College führt der Weg eines amerikanischen Studenten durch ein vierjähriges Medizinstudium, welches aus zwei Jahren Grundlagenlehre und zwei Jahren klinischer Ausbildung besteht. In dieser Überblicksarbeit werden wir uns mit einigen dieser Gemeinsamkeiten und Hauptunterschiede befassen.

## Background

Historically, Germany and the United States have had a long and close relationship in many perceivable aspects: economically, politically, culturally, as well as military. The same concept applies to science and education, partcularly in the medical field. In the late 19^th^ and early 20^th^ century, Germany was considered the pinnacle of medical education, clinical skills, and research pertaining to the human body. Numerous physicians from Germany rose to fame in that period of time, such as Alois Alzheimer [1], Emil von Behring [2], Robert Koch [3], Rudolf Virchow [4], or Albert Schweitzer [5] who shaped medicine to what it is today. The German standing in natural science attracted many international colleagues from overseas who would travel far to learn from German expertise. Their influence was far reaching, as Abraham Flexner, a German physician, has been regarded as a key inspiration in the development of medical schools in the United States [6], [7].

Nevertheless, during the second half of the 20^th^ century, the two countries appear to have drifted apart in the context of medicine. Germany employs a governmentally controlled universal multi-payer system ensuring medical health coverage for close to 100% of its citizens, whereas the United States heavily relies on insurers from the for-profit private sector. The United States has also instituted a fairly standardized 4-year medical degree (M.D. or D.O.) across their nation, which generally requires prior 3–4 years of undergraduate education with a Bachelor’s degree where the enrolled students disburse the large tuition costs. In Germany, however, the vast majority of medical schools are state and tax-funded. They encompass a fairly standardized integrated 6-year curriculum that begins directly after high school and culminates in a medical degree after successful completion of all state board exams (*Staatsexamen*).

The leading countries of Western societies constantly aspire to develop a health care system with medical schools that train doctors to deliver optimal medical care and cost effective medicine. Perhaps partly due to the formally distinct developments in these two countries, it is now the US that is considered as pioneer in structured medical education, clinical work, and scientific endeavors. German faculty and students today often seek the cooperation with American universities that are perceived by some as superior in their didactical efforts and more structured education [8]. These notions may explain why Anglo-American countries are the most popular destinations for German students to do foreign rotations, semesters, or entire research projects [9], [10].

The objetive of this report is two-fold. First, the authors want to introduce and briefly delineate the medical education systems in both countries to international readers. Secondly, a non-systematic comparative review aims to help readers understand the differences and similarities between both systems of medical schools, point out potential assets and drawbacks in each country, and ultimately fill a void in international medical literature. The intended audience for this report are primarily medical students at any stage of their training, but also young physicians or even graduating high school students who are considering time abroad during their universitary education.

## Methods

In this narrative review, the online databases MEDLINE and PubMed were searched for reports published until May 2017 that addressed medical education and associated topics in either Germany or the United States. Search terms included but were not limited to “education”, “medical school”, “medical university”, “student”, “physician”, “German(y)”, “United States”, and combinations thereof. References found within these articles were searched as well. Publications that were not published in either English or German language were excluded. The articles found in literature that were determined pertinent and up-to-date with the current subject matter were selected in this review. Additionally, the authors analyzed up-to-date legal guidelines of official public organizations, reviewed distinguished medical as well as non-medical media outlets, but also included their personal experience. All findings were stratified following the typical chronological career path of a medical student. No studies on human or animal subjects were performed.

## Medical school in Germany

### Current situation

Medical education in the Federal Republic of Germany is offered through one of the 36 medical faculties of public universities that are tax-funded through the respective states [11]. Private universities, including but not limited to the Universitaet Witten Herdecke, or the Austrian-based Paracelcus Medizinische Privatuniversitaet with a location in Nuremberg, are a small minority and are not considered within this review. Almost 80,000 students are enrolled in a medical program. Each year, almost 10,000 new students begin medical school while 6,000 successfully graduate [12]. The curriculum defining the number of classes, minimum requirements, and guidelines for examinations is designed by federal officials of legislature and subsequently written into law: *Approbationsordnung für Ärzte (ÄApprO)* of 2002 [13]. It aims to ensure that all students in Germany receive the same level of education and can later provide high quality patient care regardless of the location of medical training. The universities have the freedom, however, to execute these requirements in a fashion and order as they see fit, as long as they follow the legal guidelines [14].

### Application process

Every German resident with an advanced high school diploma (*Abitur*) or foreign equivalent is eligible to apply to a medical program. Due to the increasing popularity of spots in medical faculties, matriculation into medical school has become extremely competitive. Recent estimates demonstrate approximately 5 or more applicants per spot in public universities, depending on class and year [15]. This entire bureacratic operation that aims to ensure a fair distribution of these spots to the strongest applicants is managed by one central governmental non-profit trust called *Stiftung fuer Hochschulzulassung* [16] (formerly *Zentralstelle für die Vergabe von Studienplätzen*).

The major criterion for a succesful application and matching in one of the medical programs is the grade point average, or GPA (*Abiturschnitt*), after leaving high-school (*Gymnasium*). It is calculated based on a student’s performace during 11^th^ and 12^th^ grade of school, a brief research paper, and their final school examinations. 1.0 is considered best while 4.0 is the minimum GPA required to graduate from high school. In addition to that, a large number of universities provide bonuses for participating and passing an exam called TMS (*Test für Medizinische Studiengänge*) [17], which is similar to the American Medical College Admission Test (MCAT) and can improve a GPA up to 0.8 points depending on a student’s percentile. Furthermore, minor GPA improvements can be achieved for prior military/civil service or completion of a nursing degree apprenticeship. Certain faculties even provide additional GPA credit for superior performance in scientific classes during high school. During the application process, the high school graduates need to choose up to 6 universities they are planning to apply for and rank them in order of preference. Moving on, the Stiftung fuer Hochschulzulassung distributes the available over 9,000 spots in German medical schools threefold [18]: 20% of all spots are given to those with the highest GPAs in state rankings. 60% are accepted via the internal process of medical faculties which is based on the final GPA including bonuses and occasional personal interviews. The final 20% are admitted by the number of “waiting semesters” (*Wartesemester*), meaning those who have waited the longest since high school graduation, without enrolling in a public university, have the greatest chance for sucessful application to medical school. For the medical year of 2016/17, the GPA cutoff for the first 20% via state rankings was either 1.0 or 1.1 depending on the home state. Cutoffs for GPA with bonuses via the internal distribution process were around 1.3. Acceptance for the last group (20% of spots) via waiting list required a minimum wait of 14 semesters (7 years) since high school graduation [19]. Defining ideal admission criteria will remain a subject of ongoing debate between students, medical faculties, and politics [14].

Overall, the process is not without some intricacies. Three rounds of ranking and matching are typically needed to assign all the available spots and the last students are informed about their successful acceptance as late as October – at which point most universities have already begun orientation and first lectures.

### 2-year preclinical segment

The six year German medical curriculum consists of 2 major parts: the 2-year preclinical segment (*Vorklinik*), and the 4-year clinical segment (*Klinik)* where the final year includes rotations only *(Praktisches Jahr*) [20]. During the first four semesters of medical school the students partake in classes of basic science such as chemistry, biology, organic chemistry, physiology, physics, psychology, as well as microscopic and macroscopic anatomy. Teaching is provided by voluntary lectures, mandatory seminars and practical courses, such as the cadaver lab. Upon succesful completion of all 16 credits and a mandatory unpaid 3-month nursing internship, which is comparable to a nursing assistant in the U.S., a student is qualified to apply for the first state boards exam (*Staatsexamen*) no earlier than 2 years after starting medical school. This exam consists of two stages, a two-day multiple-choice part of 320 items [21] and a one-day oral test in small groups lead by three faculty members of anatomy, organic chemistry, and physiology.

### 4-year clinical segment

Upon passing both parts of the first exam with a grade of 4 (American equivalent: D) or better, the medical student is permitted to start his clinical segment of medical school [20]. The initial three-year segment of semesters five through ten covers all relevant clinical subjects from surgery, orthopedics to pathology, microbiology, and genetics. In recent years, many faculties have additionally started so-called interdisciplinary classes combining medical, surgical, and pathological aspects into one block based on disease entity. Exams are performed via multiple-choice – by far the most frequent type of exam, oral/practical evaluation, for example objective structured clinical encounters (OSCE), or essay-writing. In addition to the core clerkships that usually last one or two weeks each, each student must individually select 4 months of rotation during their vacation between winter and summer semesters: 2 months for in-patient care, 1 month in the out-patient setting, and 1 with a board-certified family physician. Once these criteria are fulfilled, medical students in Germany typically partake in the second state board exam after their 5^th^ year of education. It entails 320 multiple-choice questions [21] over a course of three consecutive days. In recent years, German universities have begun to implement more innovative and practical aspects into the clinical segment of medical training. This includes the use of standardized actor-patients and classes for communication skills together with colleagues from psychosomatics. Problem-based and computer-based learning are gaining the attention of faculties, too. In addition, students are now required to evaluate their lectures and courses on a regular basis, which has helped raise satisfaction with the current teaching formats [14]. Some universities have gone one step further and transition to so-called “model programs”. They employ curricula that simultaneously spread both theoretical knowledge and clinical skills from the very first day. In addition, they suggest changes to the current board exams, the division of the final year of medical school into four instead of three segments, and mandate scientific projects for all students [22]. The German Council of Sciences (*Wissenschaftsrat*), an independent counseling agency for scientific-political topics, has recently issued an official statement advising German lawmakers to expend these model programs to all public universities [23].

Next, the final year of medical school constitutes three rotations of 16 weeks each: internal medicine, surgery, and one elective outside of surgery or internal medicine. Sub-rotations within the institutions are common and this period is often also used as an opportunity for away rotations at affiliated teaching hospitals or even abroad at accredited medical universities [9]. This final year additionally serves the purpose for the medical students to get in contact with their desired departments and plan their application process for a future residency. Afterwards, the third and final state board exam takes place. This is a 4-hour oral examination similar to the oral stage of the first board exam. Overall, the minimum length of medical school in Germany is defined as 6 years and 3 months [20]. However, it is not uncommon for medical students to engange in scientific activities and in this vein, pursue an academic *“Dr. med.” *[24] degree. Depending on the underlying nature of the project, it might necessitate one or a few semesters of absence from classes. Physicians in Germany can only be addressed as “Doctor” upon submitting and defending their *“Dr. med.”* thesis in an oral exam. In the United States, however, a doctoral degree, either as M.D. or D.O., is automatically awarded upon graduation regardless of scientifc accomplishments. Upon succesful completion of all state board exams, the medical graduate can apply with the state medical association for a full medical license (*Approbation*) and bear the title “physician” (*Arzt/Ärztin*) given an inconspicuous physical exam and a clean criminal background check.

### Quality control

The highest rank of teaching at German universities is a postdoctoral lecture qualification (*Habiltation*). It is usually awarded in form of a degree of a *“Dr. habil.”* or of a “*Privatdozent”* and demands that an array of requirements must be met before suitable candidates can be considered for *Habilitation*. This status is implemented in a very standardized fashion at all public German universities but certain details may differ. Physicians needs to have a prior *“Dr. med.”* degree verifying scientific endeavors in the past. Publications in peer-reviewed journals, the candidate’s author positions in these manuscripts, certificates of continuing medical and didactic education, and numerous proofs of teaching are additional prerequisites. After habilitation, the faculty member must continue to be involved in his clinical field in terms of science and student education to maintain this high academic status [25].

Nevertheless, current trends seem to favor teaching in the practical setting and in smaller groups rather than large lectures. Due to limited numbers of academic personnel, the participation of resident physicians, fellows, and even senior students in peer-teaching sessions [26] is becoming more crucial. In these instances, faculty members with *Habilitation* need to educate and prepare their younger colleagues individually for their upcoming classes to ensure high-quality education for all medical students. Furthermore, those physicians interested in academic careers have the option to pursue graduate education to demonstrate their teaching qualification objectively. Graduate programs leading to a Master of Medical Education (MME) [23] and similar degrees are available and have shown positive impact on healthcare professionals in previous studies [27] yet their value still needs to be verified on a larger scale.

### Financial aspects

The public universities and their respective medical faculties discussed in this report are predominantly government- and thus tax-funded, as it has been the norm for many decades in Germany. After a short period of tuition charges of 500 Euros per semester (1,000 Euros per year) [28] starting around 2005 in various German states, these fees were all abolished again by the winter semester of 2014/15 [29] due to massive protests from students and the general public and shifts in the political atmosphere. Today, merely administrative fees exist ranging around 50–90 Euros per semester as well as discounted tickets provided by the universities for unlimited use of public transportation ranging from 50 to 200 Euros per semester. Generally, cost of living, particularly housing [30] in the more expensive cities, has been the major financial burden for students in Germany. For these reasons, many scholarship offers are more focused on specific student groups, such as political parties or religious communities rather than reaching out to the general student body [31]. The largest providers of collegiate financial support are the so-called *Deutschlandstipendium*, which reached merely 0.84% of all students [32] in Germany, the *Studienstiftung des deutschen Volkes* with similar scholarship figures [33], and the national *Bundesausbildungsförderungsgesetz (BAföG) *[34], that provides monetary aids up to 670 Euros per month to qualifying low-income students – half as a scholarship, half as a student loan. Altogether, only 4% of all German students received scholarships according to a recent survey [35].

### Postgraduate career: residency, research, and the private industry

Akin to many other industriliazed nations, Germany is facing the challenge of a constantly aging population requiring attention from medical professionals [36]. Unfortunately, this demand does not seem to be met by the large numbers of new physicians graduating every year. Of those that start medical school, about a third do not graduate. Of those that do, many opt for alternative careers outside of patient-care, such as research, administration, or the private industry sector. Others may only work part-time or pursue a residency abroad in the pursuit of better training, a harmonic work-life balance, or higher compensation [37]. For these reasons, Switzerland has been the number one destination for emigrating German physicians. Young physicians that decide to commence residency (*Facharztausbildung*) in Germany need to apply independently at various accredited institutions unlike during the centralized and nation-wide application for medical school. Residencies in general last a minimum of 48 to 72 months depending on specialty. An array of fellowships (*Zusatzbezeichnung*) can be obtained after succesful completion of residency. Interestingly, some smaller hospitals and their respective program directors may not have full credentials for the entire spectrum of clinical rotations to complete the specific residency, so residents may have to complete parts of their residents at different institutions. One upside to this tradition is that a residency can commence at any time of the year when there is a free resident employment offer at an accredited instritution. While there are guidelines for resident hours and compensation [38], employment length and other minor details can be negotiated individually. More competitive specialties with greater numbers of applicants may therefore have more leverage in their hiring process. Futhermore, particularly surgical specialties bear catalogues of minimum procedures that need to be performed before applying for the board certification with the state [39]. A thorough analysis of each specialty would be beyond the scope of this report. In summary, residency training in Germany is highly variable and dependant on multiple factors.

## Medical school in the United States

### History and current status

There is a total of 180 medical schools in the United States, 147 of which are allopathic [40] and 33 of which are osteopathic schools [41]. Of the allopathic schools that graduate students with an M.D. degree, 60.5% are public compared to only 20.0% of osteopathic schools providing a D.O. degree [42]. The osteopathic programs have become very similar to allopathic ones with the difference that their curriculum includes bone and joint manipulation. Considering the continuously rising popularity of careers as physicians, these osteopathic schools are quickly increasing in numbers to somewhat compensate for the rising demand [7]. Many American students, finding acceptance into US medical schools exceedingly competitive, often opt for international schools, most commonly in the Caribbean. However, detailed discussion of medical training through Caribbean medical schools is beyond the scope of this review.

The Flexner Report, written by Abraham Flexner and published in 1910, has been attributed to greatly influencing the current medical education system in the United States [43]. More specifically, the report called for higher admission and graduation standards at medical schools in addition to teaching more structured and established aspects of mainstream science and medicine. Allopathic medical schools are accredited by the Liaison Committee on Medical Education (LCME) and are sponsored by both the American Medical Association (AMA) and the Association of American Medical Colleges (AAMC), whereas osteopathic medical schools are accredited by the Commission on Osteopathic College Accreditation of the American Osteopathic Association. There has been concern in the past that the medical school curriculum places too much emphasis on the natural sciences at the expense of the psychosocial, humanistic, and professional aspects of medicine [44]. In the 1990s and 2000s, the LCME and Accreditation Council for Graduate Medical Education (ACGME) required medical schools and residency programs to teach and assess professional attributes [44].

The 4-year-long Flexner model of two years of basic science instruction followed by two years of clinical experience has been rigorously maintained. This process ensures and maintains educational rigor across institutions [45]. Pre-clinical years generally span 2 years, during which students attend didactic lectures and focus on the natural sciences. Clinical exposure is standardized to 2 years, providing students the opportunity to practice clinical sciences and patient interactions. However, similar to German tendencies, recent medical education reform has transitioned to including greater integration of clinical application and humanistic qualities of the profession earlier during training, moving away from the traditional Flexner model [46]. The transitions may include the change from large audience lectures to classes of smaller groups and the earlier implementation of clinical knowledge. Due to many federal regulations in form of Institutional Review Boards (IRB) any reforms demand time to be successfully tested [7].

### Application process

There are two paths to gaining acceptance into a medical school in the United States. The more common and traditional method entails gaining acceptance into a 4-year university and completing a Bachelor’s degree while completing 2 years of pre-medical requirements. These requirements vary by school but most commonly include one year of biology with lab, one year of general chemistry, one year of organic chemistry with lab, and one year of physics. Some schools also require coursework in behavioral and social sciences, one year of writing/English, and up to one year of mathematics. Students must also take the MCAT as a standardized test with the purpose to assess one’s capacity for the rigors of medical school.

Alternatively, high school students may enter a combined B.S./M.D. or B.A./M.D. program. These programs allow students to earn a Bachelor’s degree and then proceed directly into a medical program for a Doctor of Medicine (M.D.). One benefit to these programs is that students may forego the typical medical school admissions process that most pre-medical students undergo at the end of their undergraduate careers. Additionally, these programs are often in the form of accelerated 6- or 7-year programs (as opposed to the traditional 8) and most, but not all, relinquish the student from MCAT requirement.

Application numbers to medical schools in the United States are at an all-time high and increasing every year. Most recently, 53,029 applicants applied in 2016 and 21,025 matriculated, yielding an acceptance rate of 39.6% [47]. The rate of growth in medical school positions has not matched the rate of demand, and therefore the last several years have demonstrated an average downward trend in acceptance rate with few exceptions.

Studies have shown that scores on the MCAT have limited predictive validity for medical school performance and licensing exam measures [48]. With the understanding that objective measurements including test scores and grades are not sufficient to identify candidates who will go on to become competent and successful physicians, the medical school admissions process has moved toward a more holistic approach including increasing the weight of nonacademic data [44].

### 2 years of basic science

In recent years, the educational format in many U.S. medical schools has transitioned to including more accompanying small group learning sessions, such as problem-based learning (PBL) [49], in addition to traditional didactics, such as large lectures and medium-sized seminars. Several studies have reported that group learning in PBL may have positive effects. However, additional research is required to obtain more insight on the cognitive and emotional effects on medical students in this format.

Simulated patient encounters and improvements in simulation technology, now providing students with mannequin-robots that talk, blink, breathe, and move, have provided medical students of this era with increased opportunity to develop autonomy in a safe, realistic, yet artificial environment [45].

The two preclinical years culminate in the United States Medical Licensing Examination (USMLE) Step 1. This is the first of three steps to obtain a medical license in the United States, and it is sponsored by the Federation of State Medical Boards (FSMB) and the National Board of Medical Examiners (NBME). This test holds great weight in residency admissions, therefore medical students commonly spend large amounts of time studying on their own or in groups [50].

### 2 years of clinical rotations

The USMLE Step 1 is generally taken at the end of the second year of medical school, although there are a few exceptions. Immediately following Step 1, students generally begin their third year, marking their transition into the clinical years. The third year of medical school is structured to ensure exposure to core disciplines of medicine, including internal medicine, surgery, obstetrics and gynecology, psychiatry, and others. Students are encouraged to decide on a specialty to pursue by the beginning of their fourth year, as this time is more flexible, allowing students the opportunity to create a schedule tailored to increasing exposure to their desired specialty. At the end of the third year or beginning of the fourth, medical students take the USMLE Step 2. This exam is divided into two sections: clinical knowledge (CK) and clinical skills (CS). Step 2 CK is similar to Step 1, testing students’ knowledge on a computer-based standardized exam. Step 2 CS was introduced in 2004 as a pass/fail one-day observed series of simulated patient encounters to ensure the necessary clinical skills to be effective physicians. The test is assessed by three criteria: (1) integrated clinical-encounter (ICE) including gathering data such as history and physical exam and writing a note, (2) communication and interpersonal skills, and (3) proficiency of spoken English [51]. Students graduating with an M.D. must pass all three steps of the USMLE exams prior to practicing medicine in the United States.

### Maintaining teaching standards

Regulatory bodies as well as individual institutions have enacted policies to maintain teaching standards in U.S. medical education. In March 2013, the American Medical Association (AMA) on Medical Education approved faculty credit for teaching medical students and residents as an activity that can be certified for credit. The AMA historically offered Category 1 Credit for teaching at live Continuing Medical Education (CME) activities, and Category 2 credit for teaching medical students and residents [52]. Additional information about CME can be found at the Accreditation Council for Continuing Medical Education webpage [53]. Physicians use CME credit to demonstrate participation in educational activities to meet requirements for state medical boards, medical specialty societies, specialty boards, hospital medical staffs, the Join Commission, insurance groups, and others [52], [54]. In order to quality for AMA Category 1 or 2 Credit, the instruction must meet a list of AMA core requirements [54], [55].

Several medical schools in the U.S. require faculty physicians to regularly instruct students. Ohio State University (OSU), for example, states that the receipt of University salary entails a requirement to teach, principally to medical students, but may also be applied to the teaching of undergraduate and graduate students as well. OSU expects faculty physicians to allocate one half-day per week for teaching obligations [56]. Harvard Medical School Masters in Medical Education Program similarly places high priority on the advancement of medical education through research, skill building, and innovation, thereby seeking to transform medical education in the service of advancing the health sciences and healthcare [57].

Professionalism standards nationwide have also received much attention at medical schools, particularly during recent curricular reform. This increased attention to professionalism at the medical student level has also been attributed to propagating attention to professionalism among faculty, residents, and staff [58].

### Cost of attendance

The cost of medical school tuition in the United States has developed a reputation worldwide for being exceedingly expensive. Tuition, fees, and health insurance at public medical schools averages at $34,592 per year for residents and $58,668 for nonresidents, meaning those who are not from the same state as the school. Private medical schools cost an average of $55,534 per year for residents and $56,862 for nonresidents [59]. These figures do not include living expenses, which vary in each locale. A total of 76% of medical students graduate with educational debt [60]. Of these students with debt, the average for students graduating from public medical schools is $180,610 (median $180,000) and the average for students graduating from private schools is $203,201 (median $200,000) [60]. Additional premedical education debt, referring to undergraduate university studies, has most recently been estimated with average figures of $25,550 to $39,950 depending on college type [61]. The majority of tuition and living expenses are paid by family contribution or loans. Part-time employment is uncommon among medical students in the United States, and in many schools strictly forbidden. Few students are fortunate to receive significant scholarships to alleviate the financial burden of medical school. The significant debt of graduating medical students is considered the major burden of becoming a US physician. These costs translate to the fact that the United States operates the most expensive healthcare system in the world [7].

### Residency match or alternative career paths

Most medical school graduates pursue a residency through the National Residency Matching Program (NRMP), which is sponsored by the American Board of Medical Specialties (ABMS), the American Medical Association (AMA), the Association of American Medical Colleges, (AAMC), the American Hospital Association (AHA), and the Council of Medical Specialty Societies (CMSS). The NRMP came about in 1952 in response to dissatisfaction with a decentralized and highly competitive market in securing a residency position. This program standardizes the entire application process across nearly all specialties and somewhat mitigates the bureaucratic difficulties for foreign medical graduates. Upon successful completion of all USMLE exams and application for the Educational Commission for Foreign Medical Graduates (ECFMG) diploma, an international medical graduate can apply for residency through the NRMP without large obstacles. In contrast, obtaining a license in Germany as a non-EU citizen would require individual and tedious communication with one of the state medical associations (*Landesärztekammer*). Two specialties, including urology and ophthalmology, utilize their own separate matching process outside of the NRMP. Both urology and ophthalmology do, however, use the NRMP in securing preliminary or transitional training years. The American Urological Association (AUA) dictates the matching process for urology applicants, and the San Francisco Match (SF Match) is responsible for the matching process in ophthalmology.

Medical students apply to the residencies in their desired specialty during their fourth year. The application cycle is standardized using one of the three matching processes listed above, depending on specialty. Strict restrictions and regulations regarding contact between applicants and programs is enforced to maintain the integrity of the matching process and reduce inequities in securing a residency position.

## Discussion: Differences and overlaps

A society that aims to create brilliant physicians requires a brilliant educational system. Even though Germany and the United States are both wealthy and highly industrialized countries providing outstanding healthcare of the most recent standards, engage in medical and scientific knowledge exchange, conduct research together [62], and develop modern guidelines for patient care, their approaches to training medical students are significantly different (Table 1 (Tab. 1)).

The first major discrepancy is the structure of the academic process between high school and graduating as medical doctor. If successful in the highly competitive application process to medical schools, German students enjoy the simplicity of an integrated 6-year program that allows them to focus completely on their studies, clinical rotations, or any research activities knowing that a medical degree is guaranteed if all credentials are successfully completed. On the other hand, the United States rather employs a two-stage process. American undergraduate students initially require a Bachelor’s degree that may or may not involve participation in classes unrelated to the medical field. Although certain pre-medical prerequisite courses are required to apply to medical school, these only account for 2 years of the typical 4 required for a Bachelor’s degree. Next, it is necessary to take the MCAT exam and once again go through the stress and financial burdens of the application and interview process to medical school. The second significant distinction in medical education is of monetary manner. Despite recurring public debates about the high levels of tuition costs in the United States, these fees have been steadily increasing in a manner that is exceeding inflation. Interestingly, this financial obstacle does not seem to impact the popularity of medical school programs since student applications remain high. Large tuition loans are typically paid back after residency when six-digit physician salaries are norm and taxes lower than in Germany [63]. 

Nevertheless, Germany and the USA also share similarities. In both countries, first year medical students run through a two year basic science program before proceeding to the clinical courses of medicine. On their way to a medical degree, students take standardized board examinations. These overlaps in the structure medical education are often used by students to schedule away rotations and learn about other countries, healthcare systems, and teaching patterns. However, traveling abroad for electives seems to be more common for German than American students who may be limited in their freedom of permitted clinical rotations.

Ebrahimi-Fakhari and colleagues from Germany, for instance, described their final year experiences in large academic US institutions. They praised the structured rotations, clear roles in a medical team of attendings and residents, the handling of own patients under supervision, and the overall positive teaching culture providing immediate feedback. The authors encouraged such an environment to German universities and promoted the extension of these international partnerships [9]. A number of such alliances currently exists: the Ludwig-Maximilian University in Munich keeps up a partnership to Harvard University [8], the University of Heidelberg offers 4^th^ year rotations at different US institutions [64], and the University of Rostock has a strong cooperation with the East Tennessee State University [65]. In addition, the Technical University of Munich regularly hosts a course of highly motivated medical students in form of a case discussion round and with participation of faculty and M.D./Ph.D. students from Weill Cornell Medicine [66]. In a highly globalized world, it is important to maintain these international relations and learn from each other to preserve and strengthen healthcare systems. These exchange programs and collaborations facilitate the often gruesome process of application and credentialing that students undergo when trying to expand their exposure to other institutions on clinical rotations or research projects. Having access – even only for a few months – and having gone through an elite medical training abroad delivers a competitive advantage to each single medical student. German students who are well-known for their willingness to travel abroad for rotations may therefore explain the current trends in doctor migration. Germany has suffered from significant emigration of highly skilled physicians for many years – a deficit that has only partially been compensated by immigration, especially from countries of Eastern Europe where the standard of training may be suboptimal [67], [68]. In contrast, the US has recorded a continuous growth in numbers of foreign medical graduates applying for residency while physician emigration is quasi unheard of [69].

## Conclusion

In spite of common cultural, economic, and political interests between Germany and the United States of America, there are numerous differences in their educational approaches to teaching medical students. Both systems strive to train doctors to deliver optimal medical care in a fast-changing medical world. By using different approaches, such as different class sizes, problem-based and case-based learning versus a more generalized way of teaching, they differ. Nevertheless, at certain points in medical training, both these systems overlap: these are opportune chances for clinical and scientific exchange. Strong partnerships between specific universities ease the burdens that students face in their attempt to apply for such away programs abroad. We are hopeful that these prospects will continue to grow in near future, thereby fostering intercultural collaboration, exchange of knowledge, and ultimately advancement of healthcare. We further encourage the German and American medical schools to keep up their alliances across the Atlantic Ocean.

## Notes

### Conflicts of interest, financial disclosures, and source of funding

None of the authors, nor their close family members, have a financial interest in any of the products, devices, or drugs mentioned in this manuscript. Furthermore, the authors declare that no commercial associations or financial disclosures exist that might pose or create a conflict of interest with information presented in this manuscript. No funding was received for the work presented in this manuscript.

### About the authors

D.Z. graduated from the Technical University Munich in 2015 and is a plastic surgery research fellow in Texas.

K.T.J. graduated from the University of California, San Diego in 2017 and is a plastic surgery resident in California.

J.G.N. graduated from the Technical University Munich in 2014 and is an internal medicine resident in Missouri.

B.G. is a US-trained internist, immunologist, and oncologist. He is the director emeritus of the Institute of Molecular Immunology & Experimental Oncology at the Technical University Munich in Munich, Germany and was in charge of the TUM – Weill Cornell student exchange program.

## Figures and Tables

**Table 1:**
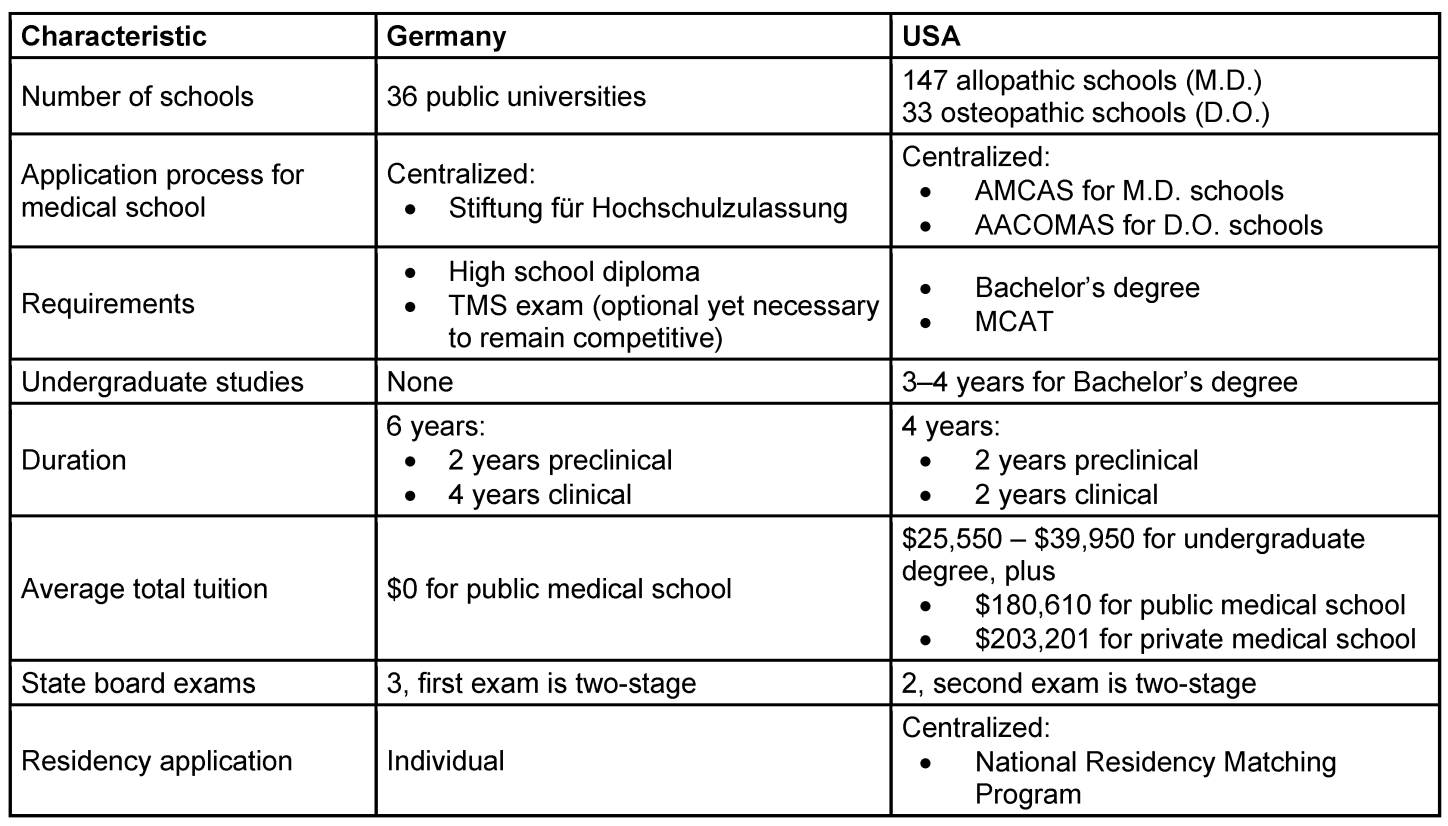
Comparison of major characteristics of medical programs in Germany and the United States
